# The Relationship Between Malocclusion and Periodontal Health in Children and Adolescents: A Systematic Review and Meta-Analysis

**DOI:** 10.3390/jcm15031155

**Published:** 2026-02-02

**Authors:** Liliana Szyszka-Sommerfeld, Monika Machoy-Rakoczy, Alla Belova, Mariusz Lipski, Laurentia Schuster, Till Dammaschke, Agata Budzyńska, Jacek Świtała, Andżelika Warcholak-Grzeszewska, Krzysztof Woźniak, Niccolò Giuseppe Armogida, Gianrico Spagnuolo, Stefan-Ioan Stratul, Marius Boariu

**Affiliations:** 1 Laboratory for Propaedeutics of Orthodontics and Facial Congenital Defects, Chair of Maxillofacial Orthopaedics and Orthodontics, Pomeranian Medical University in Szczecin, 70-111 Szczecin, Poland; 2 Department of Periodontology, Pomeranian Medical University in Szczecin, 70-111 Szczecin, Poland; 3 University Clinic of Periodontology, Faculty of Dental Medicine, Anton Sculean Research Center for Periodontal and Peri-Implant Diseases, “Victor Babes” University of Medicine and Pharmacy, 300041 Timişoara, Romania; alla.belova@umft.ro; 4 Department of Preclinical Conservative Dentistry and Preclinical Endodontics, Pomeranian Medical University in Szczecin, 70-111 Szczecin, Poland; 5 Department of Periodontology and Operative Dentistry, University in Münster, 48149 Münster, Germanytillda@uni-muenster.de (T.D.); 6 Department of Maxillofacial Orthopaedics and Orthodontics, Pomeranian Medical University in Szczecin, 70-111 Szczecin, Poland; 7 Department of Endodontic Surgery, Pomeranian Medical University in Szczecin, 70-111 Szczecin, Poland; 8 Department of Neurosciences, Reproductive and Odontostomatological Sciences, University of Naples “Federico II”, 80131 Naples, Italygspagnuo@unina.it (G.S.); 9 University Clinic of Endodontics, Faculty of Dental Medicine, TADERP Research Center, “Victor Babes” University of Medicine and Pharmacy, 300041 Timişoara, Romania; boariu.marius@umft.ro

**Keywords:** malocclusion, periodontal indices, periodontal health, gingivitis, dental plaque

## Abstract

**Background/Objectives:** Evidence regarding the effect of malocclusion on periodontal health is contradictory. This systematic review with meta-analysis seeks to summarize the available scientific evidence on the relationship between malocclusion and periodontal health in children and adolescents. **Methods**: A review of four electronic databases (PubMed, Scopus, Embase, and Web of Science) was performed. Observational studies were included if they investigated the link between malocclusion and periodontal health in children and adolescents. The quality of the studies included in the review was determined using the Newcastle–Ottawa Scale (NOS). A meta-analysis was conducted on binary outcomes using random-effect models. The Grading Recommendations Assessment, Development and Evaluation (GRADE) tool was used to determine the certainty of the evidence for each outcome. **Results**: The initial search yielded 774 potential articles. Nineteen articles were selected for the final qualitative analysis, and four of these were included in the meta-analysis. Certain malocclusion traits appear to be associated with less favorable periodontal health indicators in children and adolescents. Quantitative synthesis restricted to studies using the Gingival Bleeding Index (GBI) suggests that malpositioned teeth, such as crowding or lack of spacing, and Class II or Class III molar relationships may be associated with a higher odds of gingivitis in individuals under 18 years of age. The overall quality of evidence of the studies was very low, according to the GRADE criteria. **Conclusions**: Observational cross-sectional evidence of very low certainty suggests an association between certain malocclusions (crowding, lack of spacing, Class II or Class III molar relationships) and increased odds of gingivitis in children and adolescents. Evidence regarding dental plaque accumulation is inconsistent and limited by substantial heterogeneity across studies. Causality cannot be inferred, and further high-quality longitudinal studies are required.

## 1. Introduction

Besides dental caries and periodontal disease, malocclusion ranks as a significant oral health concern. Malocclusion affects approximately 56% of individuals under 18 globally [1,2]. Dental irregularities can lead to various issues, including periodontal problems, difficulties with oral functions like chewing, swallowing, and speaking, pain from temporomandibular disorders (TMD), and psychological distress from an unappealing smile or facial appearance [2,3,4,5,6]. Similarly, periodontal diseases pose a significant worldwide public health issue, prevalent in both wealthy and developing nations [7,8]. Gingival bleeding is the most widespread symptom of periodontal diseases, particularly among children and teenagers [8]. While periodontitis is most common among older people and its severity escalates with age, adolescents tend to show more bleeding upon probing than adults or the elderly [9].

Evidence regarding the effect of malocclusion on dental caries and periodontal health is contradictory. Specific forms of malocclusion, like dental crowding, may impede effective oral hygiene, negatively impacting oral health [10]. The challenges of cleaning crowded teeth lead to increased plaque buildup, which can contribute to the development of dental caries and periodontal disease [11,12,13]. Some research indicates a high rate of gingivitis among children with misaligned teeth [14,15]. It was discovered that certain types of malocclusions, such as crowding, extreme overjet, and crossbite, might raise the risk of periodontal disease [16]. Likewise, in another study, a potential link between malocclusion and periodontal disease was also noted [17,18]. A possible connection between crowded anterior teeth, poor oral hygiene, and dental caries in adolescents aged 15–19 was discussed, suggesting this link should be considered a caries risk factor [19]. Furthermore, it was found that anterior crowding was linked to a higher Community Periodontal Index of Treatment Needs (CPITN) score [20]. Conversely, in another study, no significant relationship between malocclusion and dental caries or oral hygiene in children could be shown. However, the authors did find a link between malocclusion in the form of increased overjet and anterior open bite and gingivitis, though no correlation was found between crowding and gingival health [6]. In a separate study, a tenuous link between malocclusion and periodontal indices was found, though a cause-and-effect relationship could not be established between malocclusion and gingivitis [21]. Furthermore, no correlation was found between the necessity for orthodontic treatment and periodontal status as measured by CPITN [22,23]. A recent meta-analysis showed that there is an absence of published evidence regarding the effects of malocclusion on oral health [24].

Due to the above cited studies, this systematic review and meta-analysis seek to summarize the available scientific evidence on the relationship between malocclusion and periodontal health in children and adolescents, as the existing research on this topic presents conflicting findings. Given the significance of these oral health issues in young people, a comprehensive overview of the existing studies is necessary. The main aim of this paper is to assess the periodontal health status expressed by oral hygiene/dental plaque accumulation, gingival and periodontal conditions in children and adolescents with malocclusion when compared with children and adolescents without malocclusion. The central hypothesis of this review is that malocclusion might influence the periodontal status of individuals under the age of 18. The primary endpoint of this systematic review and meta-analysis was the association between malocclusion and gingivitis in children and adolescents, assessed using validated gingival indices (Gingival Index—GI, Gingival Bleeding Index—GBI, bleeding on probing—BOP, gingival bleeding—GB). The secondary endpoints included the relationship between malocclusion and dental plaque accumulation (Approximal Plaque Index—API, Simplified Oral Hygiene Index—OHI-S, Plaque Index—PI, Visual Plaque Index—VPI) and periodontal treatment needs, assessed using the CPITN/CPI (Community Periodontal Index of Treatment Needs/Community Periodontal Index).

## 2. Materials and Methods

This systematic review with meta-analysis was officially recorded with the PROSPERO International Prospective Register of Systematic Reviews, under the registration number CRD420251087588. The study was carried out following the directives outlined in the “Preferred Reporting Items for Systematic Reviews and Meta-Analyses” (PRISMA) guidelines [25] (Supplementary Materials, PRISMA 2020 checklist). The primary and secondary endpoints were defined a priori. Gingivitis-related outcomes constituted the primary endpoint, while dental plaque indices and CPITN/CPI outcomes were considered secondary endpoints.

### 2.1. Search Strategy

A review of four electronic databases (PubMed, Scopus, Embase, and Web of Science) was performed by two independent reviewers (L.S-S. and M.M.-R.), using the following keywords, first designed for PubMed (“Periodontal status” OR “Periodontal health” OR “Periodontal indices” OR “Dental plaque”) AND (“Malocclusion”) AND (“Children” OR “Adolescents”). The review strategy is described in Table 1.

The literature search included all available publications without any date restrictions. The search was limited to articles published in English and also incorporated gray literature sources like Google Scholar. Additional eligible articles were identified by hand searching the retrieved literature. This process was conducted impartially to ensure all relevant studies were considered.

The following PI(E)COS research questions were the basis of this systematic review: “Is there a relationship between malocclusion and periodontal health status in children and adolescents?” and “Does malocclusion have an impact on the periodontal health status in children and adolescents?”

Population (P): Children and adolescents with malocclusion (irrespective of their gender and ethnicity).

Intervention/Exposure (I, E): Malocclusion, irrespective of its type and severity, and the methods (indices) used in its diagnosis.

Comparison/control group (C): Children and adolescents without malocclusion.

Outcomes (O): Correlation between malocclusion and periodontal health status expressed by periodontal indices/parameters regarding oral hygiene/dental plaque, gingivitis, and periodontal disease.

Study design (S): Observational studies.

### 2.2. Eligibility Criteria

Observational studies were included if they investigated the link between malocclusion and periodontal health in children and adolescents. Studies were excluded if participants had a craniofacial syndrome or a history of craniomaxillofacial surgery. There were no restrictions on the publication date of the articles.

The exclusion criteria were as follows: studies that did not directly assess the relationship between malocclusion and periodontal health, articles that included adults, who had been diagnosed with craniofacial syndrome and/or had undergo surgical interventions in the head and neck area, as well as studies with an ineligible study design, e.g., case reports, systematic or literature reviews, animal studies, or unpublished data, as well as studies in language other than English.

### 2.3. Data Extraction

Following the removal of duplicates, two independent reviewers (L.S.-S. and M.M.-R.) initially screened the titles and abstracts of the remaining studies to identify those that might be eligible. Subsequently, the full texts of these selected papers were meticulously analyzed against the predefined eligibility criteria. At each stage of the screening process, the reviewers worked independently. Disagreements were resolved through discussions between the two authors and an additional reviewer (A.B.). Throughout this process, the following data were extracted: study characteristics (author, year, country, design), participant details (age, gender ratio, total number), intervention type, outcomes (periodontal indices), and key results with statistical data. To facilitate comparative analysis, the final reviewer used spreadsheets developed according to Cochrane Collaboration guidelines. The inter-reviewer agreement was measured using Cohen’s Kappa statistics.

### 2.4. Quality Assessment

The quality of the studies included in the review was determined using the Newcastle–Ottawa Scale (NOS) [26] adapted for use in cross-sectional studies [27]. This tool was applied to assess study quality in three domains featuring seven items, based on the following star system: selection (4 items and a maximum of 5 stars), comparability (1 item and a maximum of 2 stars), and outcome (2 items and a maximum of 3 stars). According to this assessment, the overall study quality was deemed high (>7), moderate (5–7), or low (<5). The quality assessment process was carried out independently by two reviewers (L.S.-S. and M.M.-R.) after engaging in discussions and consultations with a third author (A.B.) to resolve any uncertainties or disagreements. The level of agreement between the reviewers was quantified by calculating Cohen’s Kappa coefficient.

### 2.5. Data Analysis

The extracted data were analyzed both qualitatively and quantitatively. A narrative synthesis was performed to describe variations in methodology, interventions, and outcomes across the studies.

A meta-analysis was conducted on binary outcomes using random-effect models (DerSimonian–Laird) using the Stata 11.0 software (StataCorp, College Station, TX, USA). For gingivitis outcomes, quantitative synthesis was restricted to studies using the Ainamo and Bay Gingival Bleeding Index (GBI) to ensure methodological homogeneity. Studies using other bleeding-related instruments (e.g., BOP, GI, or GB) were summarized narratively. For each study, 2 × 2 contingency tables were constructed based on the presence or absence of malocclusion and dichotomized periodontal outcomes. Odds ratios (ORs) with corresponding 95% confidence intervals (CIs) were calculated for each outcome. When zero cells occurred in 2 × 2 contingency tables, a continuity correction of 0.5 was applied. Pooled ORs were estimated using a random-effects model (DerSimonian–Laird method) to account for between-study variability. Statistical significance was set at *p* < 0.05. Heterogeneity was assessed using the I^2^ statistic and interpreted as low (<25%), moderate (25–50%), substantial (50–75%), or considerable (>75%). Sensitivity analyses were conducted by sequential exclusion of individual studies to explore sources of heterogeneity. For meta-analyses including four or fewer studies, sensitivity analyses using the Hartung–Knapp adjustment and restricted maximum likelihood (REML) estimator were conducted. Due to the limited number of included studies (*n* < 10), formal assessment of publication bias (e.g., funnel plots or Egger’s test) was not performed. Forest plots were used to visually represent the meta-analysis results, showing the pooled effect size and heterogeneity. No multivariable or adjusted analyses were performed in this meta-analysis. All pooled estimates were based on unadjusted data reported in the original studies. Therefore, the possibility of residual confounding cannot be excluded, particularly with respect to oral hygiene practices, socioeconomic status, dietary habits, and age-related behavioral differences.

### 2.6. Certainty of Evidence

The Grading Recommendations Assessment, Development and Evaluation (GRADE) tool [28] was used to determine the certainty of the evidence for each outcome. This tool considers several factors, including study type, risk of bias, result consistency, directness, precision, publication bias, effect magnitude, dose–response gradient, and confounding factors. Based on the GRADE domains, the certainty of evidence was categorized as high, moderate, low, or very low.

## 3. Results

### 3.1. Search Strategy

Of the 774 articles initially found, with 313 from PubMed, 25 from Embase, 412 from Scopus, and 24 from Web of Science, 634 were kept after 119 duplicates and 21 non-English articles were removed. Next, 589 articles were discarded for not meeting the set criteria. This left 45 articles for a full-text review, of which another 26 were excluded for not being relevant to the research question. Consequently, 19 articles were selected for the final qualitative analysis, and four of these were included in the meta-analysis.

**Figure 1:**
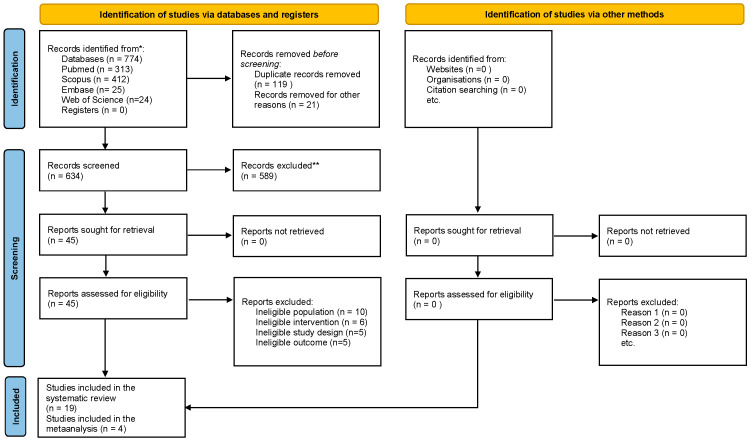
PRISMA flow diagram. * The number of records identified from each database or register searched; ** The number of records excluded by a human.

The PRISMA flow diagram visually depicts the entire search procedure (Figure 1). The reviewers demonstrated a strong level of agreement, evidenced by a high Cohen’s Kappa coefficient of 0.95.

### 3.2. Study Characteristics

Table 2 presents the main characteristics of all the studies included in the review. All of the studies were cross-sectional. Three studies were conducted in Turkey [4,20,23], two in Brazil [14,29], two in the UK [30,31] and two in India [15,22], one in Nigeria [6], one in Ecuador [7], one in Iran [10], one in Syria [13], one in the Czech Republic [12], one in Mexico [32], one in Pakistan [33], one in Poland [19], one in Jordan [34], and one in Hungary [18]. The patients’ ages ranged between 3 [29] and 19 [13,15] (Table 2).

The studies included a total of 11,954 participants. The smallest sample size was *n* = 80 [34], while the largest sample size appeared to be *n* = 1453 [4] (Table 2).

Four studies used the Index of Orthodontic Treatment Need–Dental Health Component (IOTN–DHC) [35] or Normative Need for Orthodontic Treatment (NNOT–IOTN-DHC grade 4) [10,12,13,32], while another four studies employed the Dental Aesthetic Index (DAI) [36] to classify malocclusion based on severity and the need for treatment [4,6,7,14]. Two studies used the Treatment Priority Index (TPI) [37] scores [22,23]. Three studies assessed irregularity of the teeth [30,31,34], and six studies assessed crowding and spacing [14,15,18,19,29,33] (some of them with the assessment of other types of malocclusions [15,29,33]). Three studies used the Angle classification to assess the molar relationship [15,20,33] (Table 2).

Regarding periodontal health indices, the most analyzed indices were the Oral Hygiene Index (OHI-S) [6,10,13,32], the Community Periodontal Index of Treatment Needs (CPITN) [7,20,23,33], and the Gingival Index (GI) [6,10,12,34]. Two studies used the Gingival Bleeding Index (GBI) [15,29], and another two assessed gingivitis by the presence of gingival bleeding (GB) [14,34] or bleeding on probing (BOP) [7,30]. Three studies assessed oral hygiene by means of visual examination of the presence or absence of dental plaque (VPI) [4,18,31], while another three studies used the Plaque Index (PI) [30,31,34]. One study assessed oral hygiene via the Approximal Plaque Index (API) [19] (Table 2). Table 3 presents the details on outcomes of the studies included in the review.

**Table 2:** Main characteristics of the studies included in the review.

Study, Year	Country	Study Design	Participants	Age	Gender	Malocclusion/Indices	Periodontal Indices/Parameters
Abu Alhaija & Al.-Wahadni, 2006 [34]	Jordan	CS	*n* = 80 students included and analyzed	Mean age of 12.38 ± 0.75	*n* = 39 F and *n* = 41 M	Irregularity of the lower incisor teeth (amount of spacing, mesiodistal overlap and labiolingual displacement for each of the 5 contact areas)	PI, GI, PD
Addy et al., 1988 [31]	UK	CS	*n* = 1015 schoolchildren analyzed (*n* = 3420 screened, *n* = 1018 included)	11.5–12.5	NR	STRAIT Index (irregularity of teeth)	GB, PI
Ashley et al., 1998 [30]	UK	CS	*n* = 201 schoolchildren included and analyzed (*n* = 213 screened, *n* = 12 excluded for reasons: *n* = 5 were absent from school and *n* = 7 were under orthodontic treatment)	11–14	*n* = 86 F and *n* = 115 M	Irregularity of the incisor teeth (spacing, mesiodistal overlap, labiolingual displacement)	Gingival redness and BOP, PI
Buczkowska-Radlińska et al., 2012 [19]	Poland	CS	*n* = 225 preschoolers and schoolchildren included and analyzed (*n* = 300 screened, *n* = 75 excluded for reasons: lack of consent or orthodontic treatment prior to exam)	3–19	NR	Anterior crowded teeth	API
Cortelazzi et al., 2008 [14]	Brazil	CS	*n* = 728 preschoolers included (*n* = 814 screened, *n* = 86 excluded for reasons: *n* = 31 lack of consent, *n* = 55 absent on the examination day)	5	*n* = 366 (50.3%) M and *n* = 362 (49.7%) F	DAI (crowding and spacing) (Cons et al., 1986) [36]	GB
Feldens et al., 2006 [29]	Brazil	CS	*n* = 490 included and analyzed	3–5	*n* = 230 (47%) F and *n* = 260 (53%) M	Spacing in anterior teeth, anterior open bite, posterior crossbite	VPI, GBI
Fernandez-Riveiro et al., 2021 [4]	Turkey	CS	*n* = 1453 schoolchildren included (*n* = 1843 screened, *n* = 15 excluded due to absence on the day of exam and *n*= 374 due to the presence of orthodontic treatment)	12–15	*n* = 689 M and *n* = 764 F	DAI (Cons et al., 1986) [36]	VPI
Gabris et al., 2006 [18]	Hungary	CS	*n* = 483 adolescents from secondary schools included	16–18	*n* = 289 F and *n* = 194 M	Crowding and spacing	VPI
Goel et al., 2018 [22]	India	CS	*n* = 400 included	11–14	*n* = 230 M and *n* = 170 F	TPI (Grainger, 1967) [37]	CPITN
Jafari et al., 2024 [10]	Iran	CS	*n* = 306 schoolchildren included	10–16	NR	IOTN-DHC (Brook and Shaw, 1989) [35]	GI, OHI-S
Kolawole & Folayan, 2019 [6]	Nigeria	CS	*n* = 495 included and analyzed (*n* = 503 recruited, *n* = 8 excluded due to the incomplete data)	6–12	*n* = 242 (48.9%) M and *n* = 253 (51.1%) F	DAI (Cons et al., 1986) [36]	GI, OHI-S
Kukletova et al., 2012 [12]	Czech Republic	CS	*n* = 780 participants who were referred to the clinics included (*n* = 900 invited, *n* = 120 excluded for reason: lack of consent)	13–15	NR	IOTN (Brook, 1989) [35]	GI, PI
Medina -Vega et al., 2024 [7]	Ecuador	CS	*n* = 998 schoolchildren included and analyzed (*n* = 1100 recruited, *n* = 102 excluded for reasons: *n* = 96 lack of consent, *n* = 6 absent on the examination day and refusal to be examined)	12	NR	DAI (Cons et al., 1986) [36]	BOP, CPI
Nalcaci et al. 2012 [23]	Turkey	CS	*n* = 836 students included	11–14	*n* = 384 M and *n* = 452 F	TPI (Grainger, 1967) [37]	CPITN
Öz & Küçükeşmen, 2019 [20]	Turkey	CS	*n* = 534 children who applied to the clinic included	12–14	*n* = 233 M and *n* = 301 F	Angle classification, crowding	CPITN
Pineda et al., 2020 [32]	Mexico	CS	*n* = 424 schoolchildren included (*n* = 442 screened, *n* = 439 consent, *n* = 15 excluded because they had orthodontic appliances or had received orthodontic treatment prior to the study)	13–15	53.1% F	NNOT (IOTN-DHC grade 4)	OHI-S
Salim et al., 2021 [13]	Syria	CS	*n* = 606 participants registered as refugees in Jordan and residing in Zaatari camp included	7–19	*n* = 280 (46.2%) M and *n* = 326 (53.8%) F	IOTN-DHC (Brook and Shaw, 1989) [35]	OHI-S
Sharma et al. 2021 [15]	India	CS	*n* = 1400 included	6–19	52.3% (*n* = 732) F and 47.7% (*n* = 668) M	Normal occlusion: Properly aligned teeth (absence of crowding /spacing) with Angle’s Class 1 relationship; Malocclusion: Misaligned teeth and Angle’s Class 2 and 3 occlusion	GBI
Tariq et al., 2024 [33]	Pakistan	CS	*n* = 500 schoolchildren included	13–15	44% F and 56% M	Angle classification, overjet, overbite, crossbite, open bite, diastema, crowding, and spacing	CPITN

API—Approximal Plaque Index; BOP—bleeding on probing; CPI—Community Periodontal Index; CPITN—Community Periodontal Index of Treatment Needs; CS—cross-sectional study; GI—Gingival Index; GBI—Gingival bleeding Index; GB—gingival bleeding; OHI-S—Simplified Oral Hygiene Index; PI—Plaque Index; VPI—Visible Plaque Index; PD—pocket depth; DAI—Dental Aesthetic Index; IOTN-DHC—Index of Orthodontic Treatment Need–Dental Health Component; NNOT—Normative Need for Orthodontic Treatment; STRAIT Index–Standardized Technique for Recording Alignment of Individual Teeth; TPI—Treatment Priority Index; UK—United Kingdom; NR—not reported; F—females; M—males.

**Table 3:** The details on outcomes of the studies included in the review.

Study, Year	Outcomes
Abu Alhaija & Al-Wahadni, 2006 [34]	All subjects were examined by one examiner for oral hygiene status and periodontal condition. Each subject had alginate impressions for the lower jaw, periapical X-ray for the lower incisor teeth and clinical examination for periodontal health. The mesio-buccal, mid-buccal and disto-buccal sites together with the corresponding lingual sites on each of the 4 lower incisor teeth were assessed in each subject. Oral hygiene was evaluated by examining the dental plaque present on the lingual and labial surfaces of the lower incisor teeth, using the criteria of the plaque index (PI) of Silness and Löe. Gingival condition was evaluated for the lower incisor teeth using the criteria of the gingival index (GI) of Löe and Silness. Periodontal conditions were examined using probing pocket depth (PD) to measure the distance between the bottom of the pocket and the margin of the gingiva. Bone loss was measured from the periapical radiograph.
Addy et al., 1988 [31]	The plaque present at the gingival margin of the buccal and lingual aspects of all permanent teeth was recorded by a single examiner using the criteria of the PI of Silness and Löe. A mean plaque score was obtained for each child by summing the respective tooth scores and dividing by the number of teeth present. The maximum score was 6. The presence or absence of bleeding (Muhlemann and Son) from the buccal, mesial and lingual gingiva was noted after the gentle probing of the gingival margin for plaque. The scoring employed a simple negative or positive scheme with 0—no bleeding and 1—bleeding.
Ashley et al., 1998 [30]	Each subject was assessed by two examiners. The mesio-buccal, mid-buccal, and disto-buccal sites together with the corresponding palatal sites on each of the 8 upper and lower incisor teeth were assessed in each subject, yielding 48 sites per subject. The gingival assessment included the recording of the presence or absence of gingival redness and bleeding on probing (Sidi and Ashley). Plaque accumulation was assessed initially using modified Silness and Löe criteria where code 2 (plaque visible without probing) was the maximum score used. Subsequently, all the available plaque was collected from these sites and dry weight estimated (Ashley et al.).
Buczkowska- Radlińska et al., 2012 [19]	The dental examinations were carried out by two experienced clinicians who assessed caries, oral hygiene and tooth crowding. Oral hygiene practice was determined from the above questionnaires on tooth brushing frequency and by measuring dental plaque, using the Approximal Plaque Index (API, Lange).
Cortelazzi et al., 2008 [14]	Clinical examination was performed outdoors by a calibrated examiner. Gingivitis was evaluated by the use of the gingival alteration index for 5-year-olds according to the national survey carried out in 2002 in Brazil in which any sign of bleeding that occurred in three or more teeth during clinical examination was regarded as a positive finding. The presence of gingival bleeding was examined by carefully passing a Community Periodontal Index (CPI) probe throughout the gingival sulcus margin, following the sequence: distal, buccal, mesial, lingual.
Feldens et al., 2006 [29]	Clinical examination was performed by a single trained examiner. The visible plaque index (VPI) was calculated according to a simplified version of the Silness and Löe procedure, which recorded only the presence or absence of visible plaque. The examination consisted of assessment of 4 surfaces on each tooth: mesial, buccal, distal, and lingual. The plaque to be scored had to be visible beyond doubt. The mean plaque index values for each subject were calculated, representing the percentage of surfaces with visible plaque. The gingiva’s condition was assessed using the Ainamo and Bay gingival bleeding index (GBI), which evaluates bleeding on probing. The mean gingival index values for each subject were calculated as the percentage of surfaces with gingival bleeding. Gingivitis was defined when a child had at least one surface with bleeding on probing.
Fernandez-Riveiro et al., 2021 [4]	The oral examination was performed by the dentist, the dental hygienist filled out the clinical examination form at the same time. Oral hygiene was assessed by the variable dental plaque accumulation, with the absence/presence of dental plaque being evaluated visually by a periodontal World Health Organization (WHO) probe on the buccal surface of six teeth: first molars in both arches (16, 26, 36, 46) and upper and lower central incisors of one side (21, 41). The following four categories were listed: absence of dental plaque; plaque in the gingival border; plaque in 1/3 of the gingival border; and plaque in more than 1/3 of the gingival border.
Gabris et al., 2006 [18]	The patients were examined by two orthodontist. The visible plaque index (VPI) was defined after Ainamo and Bay but with some modification: the presence of plaque was examined only on the buccal surface.
Goel et al., 2018 [22]	One trained examiner conducted all the clinical examinations under the supervision of two experienced orthodontists, one experienced pedodontist and two experienced periodontists with an assistant recording the observations. The periodontal status was recorded using the Community Periodontal Index of Treatment Need (CPITN) scores as described by the WHO. Usually, two indicators, that is, gingival bleeding and periodontal pockets are used for the assessment of periodontal status. The periodontal pockets are not recorded in individuals below 15 years of age. Since the study population comprised only of children up to 14 years, the CPITN scores were set so that 0 = no sign of disease, 1 = gingival bleeding after gentle probing, 2 = presence of supra or subgingival calculus, and X = tooth not present. Only six-index teeth were examined.
Jafari et al., 2024 [10]	GI (Loe and Silness)—It was calculated to assess the gingival health status of the adolescents in mixed and permanent dentition periods. The pocket depth was measured at the mesial, distal, buccal, and lingual surfaces of 6 teeth (16, 12, 24, 32, 36, and 44). In case of no eruption of first premolar, primary first molar was examined instead. Sound gingiva was scored 0, slight edema and gingival discoloration was scored 1, red discoloration along with bleeding on probing was scored 2, and red discoloration, edema, ulceration, or spontaneous bleeding was scored 3. The mean of the four areas was calculated for each tooth, and the mean score was reported as GI for the respective adolescent. OHI-S (Simplified Oral Hygiene Index)—Six teeth were selected such that in the mandible, the first completely erupted molar tooth behind the second premolar at both sides was considered (which is often the first molar), and its lingual surface was examined. The same was done for the maxilla, and two bilateral molar teeth were selected, and their buccal surface was examined. Also, the labial surface of the two anterior teeth was examined, which often included maxillary right and mandibular left central incisors (if missing, the contralateral incisor would be selected). OHI-S includes two components of Debris Index (DI) and Calculus Index (CI). In DI, absence of debris was scored zero, presence of debris in less than one-third of the surface was scored 1, presence of debris covering one-third to two-thirds of the surface was scored 2, and debris covering over two-thirds of the surface was scored 3. In CI, absence of calculus was scored zero, presence of supragingival calculus covering less than one-third of the tooth surface was scored 1, supragingival calculus covering one-third to two-thirds of the surface or presence of subgingival calculus at some points was scored 2, and presence of supragingival calculus covering over two-thirds of the surface or linear subgingival calculus along the cervical margin was scored. Finally, the mean DI and CI values were summed to obtain the OHI-S score.
Kolawole & Folayan, 2019 [6]	The data were collected in the months of August and September 2013. Oral hygiene status of participants was evaluated with the OHI-S described by Greene and Vermillion. The amount of debris or calculus present on the facial or lingual surfaces of six index teeth in the primary (A, E, F, K, O, and P) in the primary and 8, 3, 14, 19, 24, and 30 in the permanent dentition was used to determine the debris and calculus index scores, from which the OHI-S score was calculated. The presence and severity of gingivitis was evaluated with the GI, as described by Löe and Silness. Changes in the gingiva in relation to the appropriate six index teeth in the primary (D, G, N, Q, K and T) in the primary and 7, 3, 12, 19, 23 and 28 in the permanent dentition were assessed. Four areas of each index tooth were scored, and the scores were summed and divided by four to give the gingival index for each tooth. The gingival index of each participant was obtained by adding the values of all index teeth and dividing by six. Gingivitis was classified as mild, moderate, or severe, with values of 0.1–1, 1.1–2, and 2.1–3, respectively. Gingivitis was dichotomized into mild gingivitis and moderate-to severe gingivitis.
Kukletova et al., 2012 [12]	The clinical assessment was carried out by one experienced dentist. Gingivitis was measured using the modified GI on teeth 16, 12, 24, 32, 36, 44. The index’s 0–3 scale evaluates gingivitis on or adjacent to 6 sides of the individual teeth. The presence of plaque and calculus was recorded according to Silness and Loe (PI) and Calculus Surface Index (CSI).
Medina-Vega et al., 2024 [7]	The data collection period was from March to May 2017. Six investigators were divided into three groups, each consisting of two examiners, two individuals responsible for taking notes, one interviewer, and one assistant. The presence and extent of periodontal conditions were assessed using Community Periodontal Index (CPI). Examiners gently inserted a periodontal probe in the sulcus of six sites per tooth (mesio-buccal, buccal, disto-buccal, disto-lingual, lingual, and mesio-lingual) of teeth 2, 8, 14, 19, 24, and 30. Regarding bleeding, each sextant was assigned a code: 0—no bleeding, 1—bleeding, X—tooth not presented, 9—tooth excluded. The same codes were used to record the presence of calculus. The examiners evaluated the sites for BOP (yes or no) and calculus (yes or no). Gingivitis was defined as the presence of BOP in at least one site.
Nalcaci et al., 2012 [23]	To assess periodontal status, the CPITN was used. Four experienced orthodontists and two experienced periodontists performed the clinical examinations. The CPITN scores were set so that 0 = healthy, 1 = bleeding on gentle probing, 2 = calculus or other plaque-retentive factors, 3 = shallow pocketing of 4–5 mm, and 4 = deep pockets of 6 mm or more.
Öz & Küçükeşmen, 2019 [20]	Data collection was done for a period from June to December, 2014. To determine periodontal status and treatment needs, the CPITN, which is recommended by the WHO, was used. The highest score was recorded for each tooth according to the CPITN criteria. The highest score was selected as the CPITN score of each individual, and periodontal treatment needs were determined.
Pineda et al., 2020 [32]	The measurements were done by an calibrated examiner. Debris and calculus were examined and assessed, with vestibular and palatal/lingual surfaces clinically rated using the OHI-S.
Salim et al., 2021 [13]	Oral hygiene status was registered using the OHI-S (a combination of the debris index and the dental calculus index to determine the status of oral hygiene). For those participants aged 5 to 6 years, labial surfaces of the 54, 64, 61, 82 and the lingual surface of 75 and 85 were assessed. For mixed dentitions the labial surface of 26 and the lingual surface of 46 were also considered. For participants with most of their permanent teeth the labial surfaces of 11, 26, 16, 31 and the lingual surfaces of 36 and 46 were examined. Examination was carried out by a prosthodontist, assisted by 2 junior dentists. A cross-sectional clinical survey was conducted from October 2019 to December 2019.
Sharma et al., 2021 [15]	Data collection was done for a period of 12 months from March 2019 to February 2020. Clinical examination of children (oral and anthropometric) was done by a single examiner in the presence of parents/guardians and oral health status was assessed through the WHO Oral Health Assessment Questionnaire (2013). Upon oral examination of children, gingival health status was recorded through Gingival Bleeding Index (GBI). To evaluate the severity of gingivitis, it was further categorized as: 1. No gingivitis: Absence of bleeding gums 2. Moderate gingivitis: Bleeding present in gums around ≤ 6 teeth. 3. Severe gingivitis: Bleeding present in gums around > 7 teeth.
Tariq et al., 2024 [33]	The examination was performed by two dental examiners. The data collection was completed between the periods from April 2021 to July 2021. For periodontal assessment CPITN probe was used. Scores of 0 to 4 were recorded for six indexed teeth. Score = X was recorded in the presence of missing indexed teeth. 0 = healthy gingiva, 1 = bleeding on probing, 2 = calculus present, 3 = shallow periodontal pockets of 4–5 mm, and 4 = deep periodontal pockets 6 mm were scored. Periodontal pockets were not recorded in under 15 years old young adolescents.

### 3.3. Relationship Between Malocclusion and Periodontal Health Results—Narrative Synthesis

Table 4 presents the principal results from each study included in the review.

#### 3.3.1. Gingivitis (GI, GBI, BOP, GB)

Children with misaligned teeth (e.g., crowding, spacing) and certain molar relationships (Class II and III) had a high incidence of gingivitis, which was measured using the GBI [15]. Furthermore, there is an association between dental crowding and spacing (DAI) and gingival bleeding (GB) [14]. There is also a link between the number of tooth contact areas with displacement and overlap and the number of sites exhibiting gingival redness and bleeding [30]. In addition, a connection between severe malocclusion (as per DAI) and BOP exists [7], as well as a correlation between malocclusion (assessed by DAI or IOTN, specifically moderate to severe treatment needs, anterior open bite, and increased overjet) and gingivitis (measured by GI) [6,12]. Likewise, gingivitis (GBI) is linked to a lack of spacing in the maxillary anterior teeth, although no such connection with malocclusion types like spacing in the mandibular anterior teeth, open bite, or crossbite was found [29]. In contrast, other studies reported no correlation between malocclusion (assessed by IOTN-DHC) or tooth irregularity and gingivitis [10,31,34] (Table 4).

#### 3.3.2. CPITN/CPI

A link between malocclusion and periodontal disease was reported, as evaluated using the CPITN index [33]. Still, there also are contradictory results. Other authors did not establish a correlation between the need for orthodontic treatment (TPI) and CPITN scores [22,23]. Similar findings regarding malocclusion based on the Angle classification were described, yet a relationship between CPITN scores and crowding of the anterior teeth was observed [20] (Table 4).

#### 3.3.3. Dental Plaque (API, OHI-S, PI, VPI)

A link between malocclusion (DAI > 25) and dental plaque in 12- and 15-year-olds was discovered [4]. A relationship between VPI and crowding and spacing in adolescents was also observed [18]. Children with no spacing in their maxillary anterior teeth had higher VPI scores, but no association between VPI scores and other variables such as spacing in the mandibular anterior teeth, open bite, or crossbite could be established [29]. There also is a correlation between the severity of orthodontic treatment needs (moderate to severe needs according to IOTN) and OHI-S scores [10,13], as well as a positive association between OHI-S, the severity of crowding, and contact point deflection. In addition, a negative correlation with the severity of spacing exists [13]. Likewise, individuals with NNOT, crowding (over 4 mm), and an increased overjet (over 6 mm) were more likely to have poor oral hygiene (OHI-S) [32]. Furthermore, there is an association between irregular teeth and PI [31]. In contrast, other studies found no difference in oral hygiene status (OHI-S) or amount of plaque (PI) between participants with and without malocclusion [6,30,34] (Table 4).

### 3.4. Results of the Meta-Analysis

The meta-analysis reviewed data from four cross-sectional studies published from 2006 to 2021, all of which examined the possible link between malocclusion and periodontal health. These studies included a total of 3767 participants, with individual sample sizes varying from 3 to 19. Two of the studies investigated gingivitis [15,29], while the other two focused on dental plaque [4,32].

**Figure 2:**
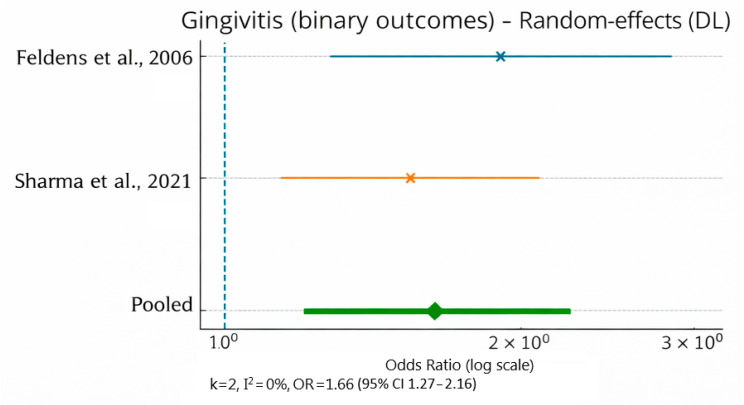
Forest plot for gingivitis based exclusively on Gingival Bleeding Index (GBI) outcomes from two cross-sectional studies (random-effects model). k—number of included studies; I^2^—Higgins’ inconsistency index indicating the percentage of total variation across studies due to heterogeneity rather than chance; DL—DerSimonian–Laird random-effects model; OR—odds ratio; CI—confidence interval [15,29].

Quantitative synthesis for gingivitis was based exclusively on two cross-sectional studies using the Ainamo and Bay Gingival Bleeding Index (GBI) [15,29]. The pooled effect size showed higher odds of gingivitis among children with malocclusion (OR 1.66, 95% CI 1.27–2.16), with no observed heterogeneity (I^2^ = 0%). This suggests that individuals with malpositioned teeth may have higher odds of gingivitis (OR = 1.66; I^2^ = 0%) (Figure 2).

**Figure 3:**
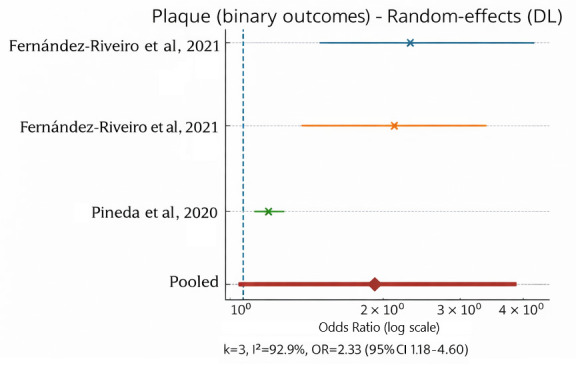
Forest plot of dental plaque outcomes from studies included in the quantitative synthesis. Due to substantial heterogeneity across studies (I^2^ = 92%), the pooled effect estimate was not interpreted, and results are presented for descriptive purposes only. k—number of included studies; I^2^—Higgins’ inconsistency index indicating the percentage of total variation across studies due to heterogeneity rather than chance; DL—DerSimonian–Laird random-effects model; OR—odds ratio; CI—confidence interval [4,32].

For dental plaque outcomes, substantial heterogeneity was observed (I^2^ = 92%), which precluded reliable interpretation of a pooled effect estimate. Heterogeneity was primarily driven by differences in plaque assessment instruments (visual plaque indices versus composite indices such as OHI-S) and age stratification across studies. Consequently, results for dental plaque are presented as a narrative synthesis supported by sensitivity analyses rather than as a summary pooled estimate (Figure 3).

### 3.5. Quality Assessment Results

Table 5 presents the findings of the quality assessment. The two reviewers showed a high degree of agreement in their assessments, with Cohen’s Kappa coefficient of 0.94. Based on the NOS assessment for cross-sectional studies, two studies were rated as high quality [6,15], ten studies were rated as moderate quality [4,7,10,13,14,19,29,31,32,33], and seven studies were rated as low quality [12,18,20,22,23,30,34].

### 3.6. Certainty of Evidence

Table 6 details the certainty of evidence regarding the association between malocclusion and periodontal indices, as assessed by GRADE. The overall quality of evidence from the studies’ evidence was “very low” for all measured outcomes.

## 4. Discussion

This systematic review and meta-analysis examines the connection between different types of malocclusions and periodontal health in children and adolescents. The qualitative analysis included 19 studies that evaluated the relationship between periodontal health and malocclusion in children and adolescents. The quantitative analysis utilized data from four studies, which collectively evaluated the link between malocclusion and dental plaque and gingivitis in 3767 individuals aged 3 to 19. The overall quality of evidence of the studies was “very low”, according to the GRADE criteria.

The results of the studies included were inconsistent, likely because of variations in the research methodologies and populations. These differences included the age range and sample size of participants, as well as the diverse indices used to evaluate oral hygiene, and gingival and periodontal health. The wide heterogeneity in the type and severity of malocclusion and the diagnostic methods used also contributed to the varied findings. Given the observational and cross-sectional nature of the included studies, as well as the lack of adjustment for potential confounders, the observed associations should not be interpreted as evidence of causality. Across studies reporting both unadjusted and adjusted estimates, adjustment for oral hygiene-related variables and socioeconomic factors generally reduced the magnitude of the association, indicating that residual confounding is likely to bias unadjusted pooled estimates away from the null. Nevertheless, certain types of malocclusion appear to be associated with less favorable periodontal health indicators in children and adolescents. However, because of the very low quality of evidence in the literature resulting from the non-randomized studies and other methodological limitations, these findings need to be carefully considered. It should be pointed out that the low quality of evidence and the cross-sectional nature of studies, as well as high heterogeneity among studies in terms of their types of interventions and/or outcomes could lead to an overestimation of the results.

In view of the above, quantitative synthesis restricted to studies using the Gingival Bleeding Index (GBI) suggests that malpositioned teeth, such as crowding or lack of spacing, and Class II or Class III molar relationships may be associated with a higher odds of gingivitis in individuals under 18 years of age [15,29]; however, causality cannot be inferred, and further high-quality longitudinal studies are required. Evidence regarding dental plaque accumulation is inconsistent [4,32]. The meta-analysis for dental plaque demonstrated very high heterogeneity, which considerably limits the interpretability of the pooled estimate. This heterogeneity appears to be largely driven by differences in plaque assessment methods (visual plaque indices versus composite oral hygiene indices such as OHI-S), age stratification, and outcome dichotomization. Although sensitivity analysis identified one study as the primary source of variability and yielded a consistent pooled effect after exclusion, these findings indicate that the impact of malocclusion on dental plaque accumulation is highly dependent on the measurement instrument used. Therefore, conclusions regarding dental plaque should be interpreted with caution and are more appropriately framed in relation to specific indices and malocclusion characteristics rather than as a uniform effect. Nevertheless, a possible link was observed between crowding and increased overjet and poor oral hygiene (OHI-S) in 13–15-year-olds [32], as well as between severe/very severe malocclusion according to DAI (DAI > 25) and dental plaque accumulation, with the absence/presence of dental plaque being evaluated visually in both 12- and 15-year-olds [4].

Most of the studies in this systematic review suggest a possible link between periodontal health and malocclusion, particularly in young people with crowding, a lack of spacing, a Class II or Class III molar relationship, anterior open bite, increased overjet, or severe anomalies (grades 4 and 5 on the IOTN scale). These occlusal issues hinder both oral hygiene and natural self-cleansing, leading to greater buildup of dental plaque, which was measured using indices like OHI, PI, and VPI [4,10,13,18,29,31,32]. An association between malocclusion and gingivitis was also observed, using assessments such as GI, GBI, GB, or BOP [6,7,12,14,15,29,30]. However, malocclusion seems to be not correlated with CPITN [20,22,23]. It should be emphasized that all the studies used a cross-sectional design, which cannot determine whether malocclusion is a cause or consequence of gingivitis and dental plaque accumulation because these variables were analyzed at the same time. Further longitudinal studies are needed to confirm the cause–effect relationship of the variables that were studied.

Tooth irregularity, crowding or spacing are important occlusal traits that have been studied extensively [13,14,15,18,29,30]. Bearing in mind the abovementioned limitations, the studies showed a possible relationship between misaligned teeth and oral hygiene [4,31,32]. It was observed that even moderate crowding was associated with a higher OHI-S score compared to mild or no crowding. The authors found a positive correlation between OHI-S and the severity of crowding in both arches, while a negative correlation was observed with the severity of spacing. Spacing was identified as a favorable condition that enhances cleansability, and consequently, periodontal health [13]. It was shown that individuals with crowding exceeding 4 mm were 99% more likely to have poor oral hygiene [32]. Likewise, a connection between VPI, crowding, and spacing in adolescents was noted, with subjects having crowding and lacking spacing showing higher VPI scores [18,29]. There is a possible link between anterior tooth crowding and dental plaque [19]. Similarly, other studies have shown that children with misaligned teeth have a high prevalence of gingivitis [14,15]. An association between crowding and gingival inflammation is suggested [30]. However, tooth irregularities appear not to be linked to significant gingival inflammation in individuals with meticulous oral hygiene practices. Similarly, no relationship between tooth irregularity and periodontal disease was found when good oral hygiene was maintained [34]. A weak but significant relationship between crowding and gingivitis has also been previously shown in adults [17]. An interesting connection between gingivitis and the lack of spacing in the maxillary anterior teeth seems to exist, but the authors did not find this same association with a lack of spacing in the mandibular anterior teeth [29]. This aligns with a previous study that showed individuals with spacing in their upper arch had less gingivitis [16]. However, this was not the case for the lower arch, where OHI-S scores did not vary significantly based on the severity of spacing, although the scores were lower for subjects with spacing [13]. This could be due to several factors, including poorer oral hygiene habits and food buildup in the mandible, as well as the reduced accessibility and manual dexterity required to effectively brush and remove plaque from these teeth [16]. It was reported that anterior crowding was associated with higher CPITN scores [20]. This positive relationship between tooth irregularity and severe gingival inflammation may stem from poorer oral hygiene, which allows for dental plaque to accumulate and leads to periodontal inflammation. However, it is important to note that crowding does not seem to affect gingivitis in patients who maintain good oral hygiene [34].

Studies that used orthodontic treatment needs indices found mixed results regarding the link between malocclusion and oral hygiene, as well as gingivitis. Some research showed that people with moderate to severe malocclusion, as classified by the IOTN, had higher OHI-S scores than those without malocclusion [10,13,32]. This was further supported by a positive correlation between IOTN-DHC scores, OHI-S, and poor oral hygiene [10]. Similarly, severe malocclusion (DAI > 25) seems to be strongly associated with dental plaque accumulation in both 12- and 15-year-olds [4]. The most common occlusal traits in this context were a maxillary irregularity of 3 mm and a maxillary overjet of 4 mm. In contrast, no difference in the oral hygiene status of participants with and without malocclusion traits according to the DAI occurred [6]. In terms of gingivitis, children with moderate to severe malocclusion had a high prevalence of gingivitis compared to those with normal occlusion [7,12]. Some studies found no link between the IOTN-DHC and gingival inflammation (GI) [10]. In contrast, a possible association between gingivitis and moderate to severe malocclusion, as well as specific features like anterior open bite and increased overjet was observed [6]. The connection between occlusal features like increased overjet and anterior open bite and gingivitis can be attributed to increased plaque accumulation caused by mouth-breathing and difficulty cleaning the teeth [38,39]. This aligns with previous studies that have shown that mouth-breathing can increase susceptibility to gingival inflammation [38,39,40]. Mouth-breathing alters the muscular forces of the tongue, cheeks, and lips. It is also believed to increase the prevalence of gingivitis due to surface dehydration and the lack of saliva’s cleansing effect [41,42,43]. The presence of nonfunctional teeth in children with an anterior open bite also contributes to the accumulation of dental plaque and debris, which leads to gingivitis [6]. Previous research has also highlighted that an increased overjet can predispose individuals to gingivitis. Both an increased overjet and anterior open bites are significantly associated with lip incompetence, leading to hyperplastic gingivitis around the upper incisors. This form of gingivitis is caused by the drying out of the oral mucosa due to the absence of lip coverage and the protective effect of saliva [41]. In contrast, an open bite and crossbite were not associated with gingivitis [29], indicating that the relationship is not consistent across all studies.

The connection between periodontal disease, as evaluated by CPITN scores, and malocclusion is unclear, with studies yielding conflicting results. A link between malocclusion and periodontal disease was found [33], while another study noted an association only with anterior segment crowding, not with the overall malocclusion classification [20]. Conversely, no correlation between the need for orthodontic treatment and CPITN scores was observed [22,23]. These mixed findings may be attributed to the nature of the CPITN index itself, which measures the periodontal treatment needs of the entire jaw, potentially obscuring localized problems with healthy areas [44]. Furthermore, the index’s limited use in research involving children may also contribute to the inconsistent results.

As mentioned earlier, some studies indicate that malocclusion and tooth irregularities do not cause significant gingival inflammation in people who practice meticulous oral hygiene [30,34,38]. This suggests that a lack of awareness about proper oral hygiene may be what leads to a higher rate of gingival bleeding. Therefore, maintaining excellent oral hygiene is considered the best way to reduce the negative impact of malocclusion on oral health. Given this, oral health strategies for young people should focus on promoting and educating them on better oral hygiene practices. When interpreting study results, it is also important to remember that the children included were of different ages and at different stages of tooth development, which can affect their awareness of oral hygiene habits. It should be remembered that other factors, such as diet, socioeconomic status, and demographics, as well as systemic diseases and orthodontic treatment can also influence periodontal health [45]. In this context, it should be emphasized that children and adolescents with systemic diseases affecting the periodontal status and/or a history of orthodontic treatment have been excluded in many studies [4,7,10,12,29,32].

### 4.1. Clinical Implications

From a clinical perspective, the findings of this systematic review and meta-analysis should be interpreted as hypothesis-generating rather than practice-changing. Although certain malocclusion traits, such as maligned teeth and Class II and Class III molar relationship may be associated with less favorable periodontal indicators in children and adolescents, the available evidence is of very low certainty, and causality cannot be inferred.

Accordingly, clinical management should continue to rely on established preventive strategies, particularly the promotion of effective oral hygiene practices and routine periodontal monitoring in children and adolescents, regardless of occlusal status. While mal-occlusion may represent a potential modifying factor for periodontal health, its independent clinical impact remains uncertain.

The possible periodontal implications of malocclusion warrant further investigation in well-designed longitudinal studies that apply standardized definitions of malocclusion and periodontal outcomes, incorporate appropriate adjustment for confounding factors, and allow assessment of temporal and causal relationships.

### 4.2. Strengths and Limitations

One strength of the present systematic review and meta-analysis is its comprehensive analysis of diverse studies, encompassing various types of malocclusion and periodontal indices. The use of the Newcastle–Ottawa Scale for quality assessment and the GRADE tool add rigor to the evaluation of study methodologies.

However, this review has several limitations that should be noted. The included studies were non-randomized clinical trials, which means the overall quality of evidence from the studies was very low according to the GRADE criteria. Differences in study populations (e.g., age, which can affect their awareness of oral hygiene habits) and methodologies (e.g., different indices used to assess occlusal features, oral hygiene, and periodontal status) could have influenced the outcomes. It should also be remembered that other factors, such as diet, socioeconomic status, and demographics, can also influence periodontal health. No multivariable or adjusted analyses were performed in this meta-analysis. All pooled estimates were based on unadjusted data reported in the original studies. Importantly, where reported, adjusted analyses consistently showed attenuation of effect sizes compared with unadjusted estimates, suggesting that residual confounding likely inflates unadjusted pooled ORs. When interpreting the results, it should be emphasized that only studies providing dichotomous 2 × 2 data suitable for odds ratio calculation were eligible for quantitative synthesis. Consequently, only four out of nineteen qualitatively analyzed studies were included in the meta-analysis. Studies reporting continuous outcomes (e.g., mean GI, PI, OHI-S values) without sufficient data for dichotomization, incomplete reporting (lack of ORs, CIs, or raw 2 × 2 data), or heterogeneous outcome definitions were excluded from pooling. While this approach increased methodological comparability, it substantially limited the number of studies included and reduced the quantitative power and generalizability of the pooled estimates. Furthermore, the review included both random samples and clinical orthodontic samples, which is another significant limitation. All the studies also used a cross-sectional design, meaning they only captured data at one specific point in time, which cannot show changes over time and cannot determine whether malocclusion is a cause or consequence of gingivitis and dental plaque accumulation because both variables were analyzed at the same time. Due to these limitations, future research should focus on long-term studies with larger participant groups and clear, consistent diagnostic criteria to better understand the topic and confirm the cause–effect relationship of the variables that were studied.

## 5. Conclusions

Based on the available evidence and within the limitations of this systematic review and meta-analysis, certain malocclusion traits appear to be associated with less favorable periodontal health indicators in children and adolescents; however, the certainty of evidence is very low. Quantitative synthesis restricted to studies using the Gingival Bleeding Index (GBI) suggests that malpositioned teeth, such as crowding or lack of spacing, and Class II or Class III molar relationships may be associated with higher odds of gingivitis in individuals under 18 years of age.

Evidence regarding the association between malocclusion and dental plaque accumulation remains inconsistent. Substantial heterogeneity across studies, driven by differences in plaque assessment instruments, age stratification, and outcome definitions, limits the interpretability of pooled estimates and precludes firm conclusions.

Importantly, all included studies were observational and cross-sectional in design, and all pooled estimates were based on unadjusted data. Therefore, causality cannot be inferred, and the observed associations may be overestimated due to residual confounding, particularly related to oral hygiene practices, socioeconomic factors, and age-related behaviors.

Overall, the current evidence should be regarded as hypothesis-generating rather than confirmatory. Further well-designed longitudinal studies using standardized definitions of malocclusion and periodontal outcomes, along with appropriate adjustment for confounding factors, are required to clarify the nature and clinical relevance of the relationship between malocclusion and periodontal health in children and adolescents.

Clinical management should continue to rely on established preventive strategies, particularly the promotion of effective oral hygiene practices and routine periodontal monitoring in children and adolescents, regardless of occlusal status. While malocclusion may represent a potential modifying factor for periodontal health, its independent clinical impact remains uncertain.

## Figures and Tables

**Table 1:** Search strategy.

Databases	Search Strategy
PubMed	(“Periodontal status” OR “Periodontal health” OR “Periodontal indices” OR “Dental plaque”) AND (“Malocclusion”) AND (“Children” OR “Adolescents”)
Scopus	TITLE-ABS-KEY (“Periodontal health” OR “Periodontal status” OR “Periodontal indices” OR “Dental plaque”) AND TITLE-ABS-KEY (“Malocclusion”) AND TITLE-ABS-KEY (“Children” OR “Adolescents”)
Embase	(“Periodontal health” OR “Periodontal status” OR “Periodontal indices” OR “Dental plaque/exp”) AND (“Malocclusion/exp”) AND (“Children” OR “Adolescents/exp”)
Web of Science	[All fields] (“Periodontal status” OR “Periodontal health” OR “Periodontal indices” OR “Dental plaque”) AND (“Malocclusion”) AND (“Children” OR “Adolescents”)

**Table 4:** Main results of the included studies.

Study, Year	Main Findings
Abu Alhaija & Al-Wahadni, 2006 [34]	No association was found between the number and type of displacement and plaque accumulation, gingivitis, attachment loss and alveolar bone level.
Addy et al., 1988 [31]	Irregular teeth retained more plaque than straight teeth. No association was found between irregular teeth and gingivitis.
Ashley et al., 1998 [30]	There was evidence for a direct relationship between the number of contact areas with tooth displacement combined with overlap and the number of sites with gingival erythema, bleeding, and profuse bleeding. There was no evidence for a relationship between labio-lingual displacement alone and gingivitis. There was an inverse relationship between the number of sites with spacing and the number of sites with bleeding, but not with the number of sites with gingival redness. There was no evidence of a relationship between incisor overlap and amount of plaque.
Buczkowska- Radlińska et al., 2012 [19]	The accumulation of dental plaque measured by API was higher in patients with anterior crowded teeth across all age groups.
Cortelazzi et al., 2008 [14]	Crowding and spacing were associated with gingival bleeding.
Feldens et al., 2006 [29]	Children without spacing in maxillary anterior teeth had a 90% higher probability of having gingivitis. The variables, such as spacing in mandibular anterior teeth, open bite, and crossbite were not associated with gingivitis.
Fernandez-Riveiro et al., 2021 [4]	Dental plaque accumulation was the most strongly associated with malocclusion (DAI > 25) in both age groups.
Gabris et al., 2006 [18]	The VPI scores for adolescents with malocclusion were higher than those of the adolescents who displayed no anomalies. A significant difference in VPI was found between subjects without crowding or with crowding in either one or two crowded segments.
Goel et al., 2018 [22]	No correlation was found between the orthodontic treatment need (TPI), and periodontal status (CPITN) scores.
Jafari et al., 2024 [10]	No correlation between malocclusion (IOTN-DHC) and GI was found. The results showed that by an increase in OHI-S score, the odds of having IOTN grade 4 compared to grade 1 increased.
Kolawole & Folayan, 2019 [6]	The mean DAI scores of participants with mild gingivitis compared with moderate/severe gingivitis differed significantly. Significantly more children with increased overjet and anterior open bite had moderate to severe gingivitis. There were no differences in the oral hygiene status (OHI-S) of participants with and without malocclusion traits.
Kukletova et al., 2012 [12]	An association was observed between GI and severity of orthodontic anomaly.
Medina-Vega et al., 2024 [7]	An association was observed between BOP and malocclusion. Children with severe or handicapping malocclusion according to DAI had a 10% higher prevalence of gingival bleeding compared to those with normal occlusion.
Nalcaci et al., 2012 [23]	No relationship was found between TPI-CPITN scores.
Öz & Küçükeşmen, 2019 [20]	The relationship between CPITN scores and malocclusion classification was not significant. The relationship between CPITN scores and crowding was significant in the anterior segment.
Pineda et al., 2020 [32]	An association was found between the presence of NNOT and poor oral hygiene (OHI-S ≥ 3). It was found that the subjects with crowding (>4 mm) were 99% more likely to present poor hygiene, which itself was 74% more likely to present in subjects with increased overjet (>6 mm).
Salim et al., 2021 [13]	Subjects with malocclusion, specifically crowding, contact point deflection and IOTN grades 3, 4 and 5 had higher scores in both arches for OHI-S than subjects without malocclusion traits. Patients with generalized spacing had lower OHI-S score than those without spacing. OHI-S was positively correlated to the severity of crowding and contact point deflection in both arches, and negatively correlated to the severity of spacing in the upper arch and in the lower arch. OHI-S was not significantly different based on the severity of lower arch spacing although those with no upper arch spacing had higher mean OHI-S than those with generalized spacing.
Sharma et al., 2021 [15]	Children with maligned teeth (crowding or spacing), and Angle’s Class 2 and 3 occlusions had a high prevalence of gingivitis. Children with properly aligned teeth in Angle’s Class 1 occlusion were 34% less affected by gingivitis than children with maligned teeth (crowded, spacing, etc.).
Tariq et al., 2024 [33]	Presence of periodontal disease was associated with malocclusion. Young adolescents with periodontal diseases were 1.57 times more likely to have malocclusion compared to young adolescents without periodontal diseases, and it was significant.

API—Approximal Plaque Index; BOP—bleeding on probing; CPITN—Community Periodontal Index of Treatment Needs; GI—Gingival Index; GBI—Gingival Bleeding Index; OHI-S—Simplified Oral Hygiene Index; PI—Plaque Index; VPI—Visible Plaque Index; IOTN-DHC—Index of Orthodontic Treatment Need–Dental Health Component; TPI—Treatment Priority Index; DAI—Dental Aesthetic Index; NNOT—Normative Need for Orthodontic Treatment.

**Table 5:** The quality assessment of the included studies.

The Quality Assessment of the Non-Randomized Studies (NOS)				
Authors, Year	Selection	Comparability	Outcome	Total Score
Abu Alhaija & Al-Wahadni, 2006 [34]	-	-	***	3
Addy et al., 1988 [31]	****	-	***	7
Ashley et al., 1998 [30]	*	-	***	4
Buczkowska-Radlińska et al., 2012 [19]	**	*	***	6
Cortelazzi et al., 2008 [14]	***	*	***	7
Feldens et al., 2006 [29]	**	*	***	6
Fernandez-Riveiro et al., 2021 [4]	***	*	***	7
Gabris et al., 2006 [18]	**	-	**	4
Goel et al., 2018 [22]	**	-	**	4
Jafari et al., 2024 [10]	*	*	***	5
Kolawole & Folayan, 2019 [6]	****	*	***	8
Kukletova et al., 2012 [12]	**	-	**	4
Medina -Vega et al., 2024 [7]	***	*	***	7
Nalcaci et al., 2012 [23]	*	-	***	4
Öz & Küçükeşmen, 2019 [20]	*	-	***	4
Pineda et al., 2020 [32]	***	*	***	7
Salim et al., 2021 [13]	***	-	***	6
Sharma et al., 2021 [15]	****	*	***	8
Tariq et al., 2024 [33]	***	*	***	7

NOS—Newcastle–Ottawa Quality Assessment Scale. This assessment covered three areas with seven criteria, utilizing a star-based system [*]: selection (4 criteria, maximum 5 stars can be awarded), comparability (1 criterion, maximum 2 stars can be awarded), and outcome (2 criteria, maximum 3 stars can be awarded). Scores/stars of 0 [-], 1 [*] or 2 [**] were awarded depending on whether the above criteria were not met, met or met using a validated method or an established model, respectively. Stars/scores in each domain (selection, comparability and outcome) indicate total points in each area [-, *, **, ***, ****]. According to the sum of these scores across all domains the overall study quality was deemed high (>7), moderate (5–7), or low (<5). Comparability (confounders investigated): family income [14,29], socioeconomic status [4,10,15,19], age [6,10,15,29,32,33], mother’s education [7,10,15,29,33], oral hygiene [6,10,15,19,32].

**Table 6:** The results of the certainty of evidence for each outcome.

Outcome	Impact	Participants (Studies)	Risk of Bias	Inconsistency	Indirectness	Imprecision	Publication Bias	Overall Certainty of Evidence *
Gingivitis (GI, GBI, BOP, GB)	Significant impact reported in 7 studies	8957 (10)	Serious ^a^	Not Serious ^b^	Not Serious ^c^	Not Serious ^d^	None ^e^	⨁◯◯◯ Very low
CPITN/CPI	Significant impact reported in 1 study	934 (4)	Serious ^a^	Not Serious ^b^	Not Serious ^c^	Not Serious ^d^	None ^e^	⨁◯◯◯ Very low
Dental plaque (API, OHI-S, PI, VPI)	Significant impact reported in 7 studies	580 (10)	Serious ^a^	Serious ^b^	Not Serious ^c^	Serious ^d^	None ^e^	⨁◯◯◯ Very low

^a^ Most of the studies included presented a moderate or low quality; ^b^ the studies included did not presented different directions of effect; ^c^ the studies provided direct evidence to the research question; ^d^ the optimal information size (≥400) was attended; ^e^ none of characteristics, such as the body of evidence consisted of only small positive studies or when studies are reported in trial registries but not published were observed. * Certainty initially rated as “low” (a body of evidence consisting of observational studies). The certainty of evidence was downgraded due to the cross-sectional design of all included studies, lack of adjustment for key confounders, and heterogeneity across outcome definitions and measurement instruments. For dental plaque outcomes, additional downgrading was applied due to substantial statistical heterogeneity. API—Approximal Plaque Index; BOP—bleeding on probing; CPI—Community Periodontal Index; CPITN—Community Periodontal Index of Treatment Needs; GI—Gingival Index; GBI—Gingival Bleeding Index; GB—gingival bleeding; OHI-S—Simplified Oral Hygiene Index; PI—Plaque Index; VPI—Visible Plaque Index; ⨁◯◯◯—very low quality of evidence of the studies.

## Data Availability

All data are available in the studies included in the review and were discussed in the present manuscript.

## Supplementary Materials

The following supporting information can be downloaded at https://www.mdpi.com/article/10.3390/jcm15031155/s1, PRISMA 2020 checklist.

## Abbreviations

The following abbreviations are used in this manuscript:

API	Approximal Plaque Index
BOP	Bleeding on Probing
CI	Confidence Interval
CPITN	Community Periodontal Index of Treatment Needs
DAI	Dental Aesthetic Index
DL	DerSimonian–Laird random-effects model
GB	Gingival Bleeding
GBI	Gingival Bleeding Index
GI	Gingival Index
GRADE	Grading Recommendations Assessment, Development and Evaluation
IOTN	Index of Orthodontic Treatment Need
NNOT	Normative Need for Orthodontic Treatment
NOS	Newcastle–Ottawa Scale
OR	Odds Ratio
OHI-S	Simplified Oral Hygiene Index
PD	Pocket depth
PI	Plaque Index
PRISMA	Preferred Reporting Items for Systematic Reviews and Meta-Analyses
PROSPERO	International Prospective Register of Systematic Reviews
STRAIT	Standardized Technique for Recording Alignment of Individual Teeth
TMD	Temporomandibular disorders
TPI	Treatment Priority Index
VPI	Visible Plaque Index
